# Similar Gastro-Intestinal Exposure to Florfenicol After Oral or Intramuscular Administration in Pigs, Leading to Resistance Selection in Commensal *Escherichia coli*

**DOI:** 10.3389/fphar.2018.01265

**Published:** 2018-11-06

**Authors:** Joren De Smet, Filip Boyen, Siska Croubels, Geertrui Rasschaert, Freddy Haesebrouck, Patrick De Backer, Mathias Devreese

**Affiliations:** ^1^Department of Pharmacology, Toxicology and Biochemistry, Faculty of Veterinary Medicine, Ghent University, Merelbeke, Belgium; ^2^Department of Pathology, Bacteriology and Poultry Diseases, Faculty of Veterinary Medicine, Ghent University, Merelbeke, Belgium; ^3^Technology and Food Science Unit, Flanders Research Institute for Agriculture, Fisheries and Food, Melle, Belgium

**Keywords:** florfenicol, administration route, intestinal concentration, *Escherichia coli*, resistance selection

## Abstract

Florfenicol, which is licensed for veterinary use only, proves to be a potent antimicrobial for treatment of respiratory disease. However, the subsequent exposure of the gut microbiota to florfenicol is not well described. Hence, the effect of various administration protocols on both plasma and gastro-intestinal florfenicol concentrations in pigs was evaluated. In field situations were simulated by application of different administration routes and dosages [single oral bolus at 10 or 5 mg/kg body weight (BW), medicated feed at 10 or 5 mg/kg BW and intramuscular injections at 15 or 30 mg/kg BW]. After intramuscular administration of 30 mg florfenicol/kg BW, gastro-intestinal concentrations of florfenicol, quantified 10 h after the last administration, were significantly elevated in comparison with the other treatment groups and ranging between 31.5 and 285.8 μg/g over the different gut segments. For the other treatment groups, the influence of dose and administration route was not significantly different. Bacteriological analysis of the fecal samples from the animals at the start of the experiment, demonstrated the presence of both florfenicol susceptible (with minimal inhibitory concentration (MIC) values of 2–16 μg/mL) and florfenicol resistant (MIC ≥ 256 μg/mL) *Escherichia coli* isolates in all treatment groups. Following, at 10 h after the last administration the susceptible *E. coli* population was eradicated in all treatment groups due to the high intestinal florfenicol concentrations measured. Moreover, selection of the resistant *E. coli* strains during treatment occurred in all groups. This is likely related to the fact that the different treatment strategies led to high gastro-intestinal concentrations albeit not reaching the high magnitude of MIC values associated with florfenicol resistance (≥256 μg/mL). Conclusively, in our experimental setup the administration route and dose alterations studied, had no influence on monitored florfenicol resistance selection in *E. coli* from the microbiota.

## Introduction

Antimicrobial resistance in bacteria is a constantly evolving phenomenon, with the extent of resistance increasing in parallel with the use of antimicrobials on a population level. The dissemination of antimicrobial resistance harbors the threat of compromising therapeutic efficacy in battling bacterial infections. Constraining these resistance issues is a crucial objective in human as well as in veterinary medicine. Current marketed antimicrobials are optimized solely based on their clinical efficacy, focussing on the target pathogen, without taking resistance selection in the commensal, non-target, microbiota into account. For instance, the intestinal microbiota (Liu et al., 2002). However, resistance in the microbiota poses a significant threat and concern for the spread of resistance in animals and also humans, according to the One Health approach (European Commission[EC], 2018). Hence, investigating the influence of the posology of antimicrobial therapy on selection of resistance in the commensal gut microbiota is an important strategy.

Florfenicol (FF), a derivate of chloramphenicol (CAP), is a broad-spectrum antimicrobial exclusively used in veterinary medicine. CAP has been banned for many years in food-producing animals, and is generally not used in human medicine because of its potential adverse effects such as aplastic anemia (Skolimowski et al., 1983). Thiamphenicol (TIA), another derivate, is available for parenteral use in case of severe infections (BCFI, 2018). Nonetheless, FF is safely applied in multiple animal species, such as swine, for respiratory tract infections. It exerts bacteriostatic activity against several bacterial species associated with porcine respiratory tract disease, including *Actinobacillus pleuropneumoniae* and *Pasteurella multocida* (Dorey et al., 2016). Acquired resistance to FF is generally very low or absent for these respiratory tract pathogens (Vanni et al., 2012). Apart from these target pathogens, the gut microbiota could also be exposed to FF during treatment. In general, FF has bacteriostatic activity against *Enterobacteriaceae* such as *Escherichia coli* and *Salmonella* Typhimurium, though often with elevated minimal inhibitory concentration (MIC) values (Syriopoulou et al., 1981). Several plasmids carrying FF resistance genes have already been associated with acquired FF resistance in *E. coli* isolates obtained from pigs (Blickwede and Schwarz, 2004) and cattle (Singer et al., 2004). Hence, antimicrobial therapy with FF could select for resistant *E. coli*, populations residing in the microbiota. Acrangioli et al. (2000) reported that all FF resistant isolates monitored, harbored *floR* resistance genes, conferring for an efflux protein belonging to the major facilitator (MF) superfamily of drug exporters (Braibant et al., 2005). Furthermore these isolates also displayed multiple drug resistance conferring to the ACSSuT (ampicillin, chloramphenicol, streptomycin, sulphonamides, and tetracyclines) genotype. Hence, FF induced selection of multi-drug resistant strains in the gut microbiota could pose a significant risk in terms of multi-drug resistance spread (Mather et al., 2013). Resistance to FF is not mediated by CAP resistance determinants, such as the enzyme chloramphenicol acetyltransferase (CAT) or the gene *cml*A (Singer et al., 2004). However, these resistance determinants are also linked to mobile genetic elements; hence co-selection of CAT resistance by use of FF is also possible (Blickwede and Schwarz, 2004).

In order to assess the effects of an antimicrobial treatment on the microbiota, intestinal concentration data could provide valuable insights, which are lacking for many molecules in the current literature. For instance data on FF intestinal concentrations and subsequent microbiota exposure, during conventional treatment strategies in pigs, are not publicly available. In general, parenteral administration allows for more accurate dosing. Furthermore, it is assumed that a parenteral administration will lead to less gastro-intestinal exposure of the microbiota, because no absorption from the gut is required. This is in contrast with oral administration of antimicrobials. Nevertheless, De Smet et al. (2017) recently reported that similar gut concentrations of sulfadiazine and trimethoprim were obtained in pigs after oral and parental treatment. The same observation was reported by Devreese et al. (2014) after administering different doses of enrofloxacin in broilers *via* oral or parenteral route.

The present study investigated the gastro-intestinal concentrations of FF in different gut segments after administering the antimicrobial through oral gavage (not used in-field) and *via* medicated feed or intramuscular (IM) injection to simulate the actual in-field situation (Callens et al., 2012). Different dosage schemes were administered to assess the possible dose-related pharmacokinetic (PK) properties of FF. Subsequently; the fecal samples from the same animals were also used to study the selective effect for FF resistance in *E. coli*, as a Gram-negative indicator bacterium for the intestinal microbiota.

## Materials and Methods

### Animal Experiment

The animal experiment was approved by the Ethical Committee of the Faculties of Veterinary Medicine and Bioscience Engineering of Ghent University (case number EC 2015-16). The experiment was conducted with 36 pigs (Topigs 20, 12 weeks old, mixed genders) randomly divided into six FF treatment groups, each with a specific dosing scheme (Table 1). The animals were group-housed (*n* = 3) in separate confinements, within the same stable, on 50/50 concrete floor/grids. Before the start of the experiment all animals were allowed an acclimatization period of 5 days. For administration through oral gavage (groups 1 and 2), Amphen^®^ oral granulated powder was used (Huvepharma NV, Antwerpen, Belgium). Groups 1 and 2 were administered an individually calculated single bolus, based on bodyweight (BW), of 5 mg FF/kg BW and 10 mg FF/kg BW, respectively, for 5 consecutive days. For the IM administrations (groups 3 and 4), Colfen^®^ (Zoetis, Zaventem, Belgium) was administered twice in total, with a 48-h-interval, as described by the manufacturer at doses of 15 mg FF/kg BW and 30 mg FF/kg BW, respectively. The injection site was located in the neck, behind the base of the ear. In groups 5 and 6, FF was administered *via* medicated feed based on the average group BWs (per confinement of 3 animals). For these administrations, Floron^®^ premix (KRKA Belgium NV, Sint-Niklaas, Belgium) was mixed into feed at an under dosage of 5 mg FF/kg BW and at conventional doses of 10 mg FF/kg BW, respectively for groups 5 and 6, again for 5 consecutive days. The animals received the complete amount of medicated feed in one administration at a fixed time point; allowing feed uptake during several hours. However, no *ad libitum* access to medicated feed was allowed, in order to estimate feed intake correctly. Blood samples (±1 mL) were collected in all treatment groups (*n* = 36) in heparin-containing vacuum tubes (Vacutest Kima, Arzergrande, Italy), through venepuncture (*vena jugularis)* at different time points (0.5, 1, 2, 3, 4, 6, 8, and 10 h) after the first administration of FF in each group. At time point 0 h and 10 h after the first administration, fecal samples were also collected in all treatment groups (*n* = 36) *via* rectal stimulation. Plastic sterile cups (40 mL) were filled, but contents were not quantified during collection. For the remaining treatment days, blood was collected at fixed time points, corresponding with the estimated time at maximal plasma concentrations (T_max_) of 2 h post-administration in all treatment groups (*n* = 36). Furthermore, blood was also collected every 10 h post-administration and also prior to the next FF administration in all treatment groups (*n* = 36). Fecal samples were collected (*via* rectal stimulation) twice daily, pre- and 10 h post-administration of FF in all treatment groups (*n* = 36) during the remainder of the experiment.

**Table 1:** Overview of the different treatment groups with mean bodyweight (BW), for the animal experiment.

Group	Administration route	Dosing scheme	Mean BW ± SD (kg)
1	PO (oral gavage)	Half dose: 5 mg FF/kg BW	28.8 ± 5.5
		Once daily, 5 days	
2	PO (oral gavage)	Conventional: 10 mg FF /kg BW	29.8 ± 4.5
		Once daily, 5 days	
3	IM (intramuscular injection)	Conventional: 15 mg FF/kg BW	34.6 ± 5.8
		2 injections, 48 h interval	
4	IM (intramuscular injection)	Double dose: 30 mg FF/kg BW	35.9 ± 5.8
		2 injections, 48 h interval	
5	PO (medicated feed)	Half dose: 5 mg FF/kg BW	30.1 ± 4.7
		Once daily, 5 days	
6	PO (medicated feed)	Conventional: 10 mg FF/kg BW	31.4 ± 3.0
		Once daily, 5 days	

*The animals were 12 weeks old, mixed genders and were group-housed (*n* = 3 per confinement) on 50/50 concrete floor/grids*.

On day three and five after initiating treatment, the animals of the IM and oral groups, respectively were euthanized, at 10 h after the last administration. Euthanasia was exerted with an intra-cardiac injection of sodium pentobarbital 20% (Kela Veterinaria, Sint-Niklaas, Belgium) after anaesthesia with a mix of 0.3 mg/kg BW xylazine (Xyl-M^®^, V.M.D. Vet, Arendonk, Belgium) and 15 mg/kg BW tiletamine-zolazepam (Zoletil 100^®^, Vibrac, Barneveld, Netherlands). Intestinal contents (luminal) were collected from different gut segments (duodenum, mid-jejunum, ileum, cecum, mid-colon, and rectum). The contents were sampled in a qualitative manner by use of sterile plastic beakers, and each sample was weighed. All blood samples were centrifuged (2851 × *g*, 10 min, 4°C) and plasma was collected and stored at ≤ -15°C for a maximum of 8 weeks until analysis for FF concentration. All fecal samples and intestinal contents were lyophilised for 48 h consecutively and weighed again afterward, and were manually grounded homogenously and stored at ≤ -80°C for a maximum of 20 weeks until quantitative and bacteriological analysis.

### Analysis of Florfenicol in Plasma and Intestinal Content

#### Chemicals and Reagents

Analytical grade Ultra Performance Liquid Chromatography (UPLC) solvents were used: acetonitrile (ACN), methanol (MeOH) and water (H_2_O) from Fisher Scientific (Erembodegem, Belgium), ethyl acetate and glacial acetic acid from VWR (Leuven, Belgium). The analytical standard of FF and internal standard thiamphenicol (TIA) were purchased from Sigma-Aldrich (Diegem, Belgium). Standard stock solutions of 1.0 mg/mL were prepared in a H_2_O/MeOH solution (50/50 v/v) and were stored airtight and protected from light at 4–8°C for a maximal period of 3 weeks. Phosphate-buffered saline (PBS) was obtained from Sigma-Aldrich (Diegem), and sodium hydroxide (NaOH) from VWR (Leuven).

#### Sample Preparation

The fecal and intestinal samples were lyophilised. The loss-on-drying from each sample was compensated for based on the weights of the samples pre -and post lyophilisation, determined by use of an analytical balance. These samples were then weighed for quantitative analysis (1.0 g) and diluted 10-fold in PBS. Following, sample treatment for fecal and plasma samples was based on liquid-liquid extraction (LLE). Briefly, 25.0 μL of the internal standard solution (TIA, 100.0 μg/mL) and 100.0 μL of a 1M NaOH solution (pH 9 for optimal extraction yield) were added to each of the fecal and intestinal samples. Next, LLE was performed by adding 6 mL ethyl acetate. After roller-mixing (10 min) and centrifugation (2851 × *g*, 10 min), the supernatant was separated and dried under nitrogen flow (40 ± 2°C). The extract was reconstituted using 500.0 μL of a 90/10 v/v H_2_O/ACN solution. Processing of plasma samples was very similar; 25.0 μL of the internal standard (TIA, 50.0 μg/mL) and 25.0 μL of a 1M NaOH solution were added to the plasma aliquot of 250 μL, 3 mL ethyl acetate was used for extraction. Reconstitution was achieved with 250.0 μL of a 90/10 v/v H_2_O/ACN solution.

#### UPLC-PDA Analysis

Liquid chromatography was performed on an Acquity UPLC H-Class system (Waters NV, Zellik, Belgium). Chromatographic separation was achieved using an Acquity UPLC C18 column (2.1 mm × 50 mm, d.p.: 1.7 μm) in combination with an Acquity UPLC VanGuard pre-column (2.1 mm × 5 mm, d.p.: 1.7 μm), both obtained from Waters NV (Zellik, Belgium). Column oven temperature was 45.0°C and the autosampler tray was kept at 8.0°C. Mobile phases for chromatographic separation consisted of 0.1% glacial acetic acid in H_2_O (A) and ACN (B). The following gradient elution program was applied: 0–3.75 min (90% A, 10% B), 3.75–5.5 min (linear gradient to 40% A, 60% B), 5.5–6.0 min (linear gradient to 5% A, 95% B), 6.0–8.0 min (5% A, 95% B), 8.0–8.30 min (linear gradient to 85% A, 15% B), 8.30–11.00 min (90% A, 10% B). Flow rate was 400 μL/min. For detection, an Acquity UPLC Photo Diode Array (PDA) detector (Waters NV, Zellik, Belgium) was used. System parameters for Ultra-Violet (UV) detection were optimized using working solutions, i.e., 0.1 μg/mL FF, and the internal standard TIA, in a H_2_O/ACN mixture (90/10 v/v). For the quantification of FF, a wavelength of 223 nm was selected for maximal absorbance, after measuring a full spectrum. The analytical method was validated based on different parameters (precision, accuracy, limit of detection (LOD), limit of quantification (LOQ), and goodness-of-fit) using matrix-matched calibrator and quality control samples. Validation was exerted according to De Baere et al. (2012).

### Pharmacokinetic Analysis

Phoenix^®^ WinNonlin^®^ 6.3 (Pharsight-Certara, Princeton, NJ, United States) was used for the pharmacokinetic (PK) analysis of the data *via* non-compartmental modelling (NCA). The area under the curve (AUC) was calculated using the linear up-log down trapezoidal method. Furthermore, several PK parameters were calculated: area under the 24 h-time curve (AUC_0-24__h_), area under the 58 h- or 106 h-time-curve (AUC_0-58__h_ or AUC_0-106__h_). The AUC_0-58__h_ or AUC_0-106__h_ values were also normalized for dosage administered by dividing these values with the actual dosage (μg) administered per animal: AUC_0-58__h_/D or AUC_0-106__h_/D. The maximal plasma or fecal concentrations C_max_ and time of maximal concentration T_max_ were also calculated by NCA.

### Enumeration, Identification and Characterization of *E. coli* in Fecal Samples

The fecal samples collected at time point 0 h before treatment and at 10 h after the last treatment (58 h for IM groups, 106 h for oral groups) were used for enumeration of *E. coli*. For these analyses, samples of three pigs were randomly selected from each treatment group for testing. The freeze-dried samples were weighed (1 g), reconstituted and serially 10-fold diluted (weight-based) in sterile PBS. Next, a spiral plater (IUL S.A., Barcelona, Spain) was used for plating 40 μL of each dilution onto (i) McConkey agar n°3 (MC; Oxoid NV, Erembodegem, Belgium) and (ii) MC agar supplemented with 16 μg/mL FF (EUCAST ECOFF value of *E. coli* for FF). After drying for a few minutes, the agar plates were aerobically incubated at 37°C for at least 20 h before performing a total plate count, on the dilution with a colony density of 20–300 per plate (Sutton, 2011). Per plate up to 3 different, large and regular shaped lactose positive colonies were purified and identified by Matrix-Assisted Laser Desorption Ionization-Time-Of-Flight Mass Spectrometry (MALDI-TOF MS, Bruker Daltonik, Evere, Belgium). For this analysis, purified colonies were applied to the MALDI target plate and covered with the appropriate matrix (1 μL of α-cyano-4-hydroxycinnamic acid) as instructed by Bruker Daltonik. MBT Compass software 4.1 (Bruker Daltonik, Evere)) was used for obtaining spectra which were matched against a database of 6.120 mean spectra projections (MSP). Only score values of ≥2.000 were taken into account.

Repetitive element sequenced-based (rep)-PCR was used for determination of genetic diversity among the different collected *E. coli* isolates. The protocols and materials used were similar to Peeters et al. (2018).

### Statistical Analysis

Statistical analysis was performed by using SPSS 25.0 (IBM, Chicago, IL, United States). Plasma or intestinal concentrations and water content results were compared between the groups by means of a single-factor analysis of variance (ANOVA) under the assumption of normality and equal variances (Levene’s test). For *post-hoc* testing either Tukey was selected or Games-Howell in case of non-equal variances, in either case the significance level α was set at *p* = 0.05. All data are represented as mean ± SD, with n indicating the number of observations, unless otherwise indicated. Mean + SD is displayed for the graphical representation of the data.

## Results and Discussion

### Analytical Method Validation

The validation results for the different parameters are presented in Supplementary Tables A, B. The limit of quantification (LOQ) for FF was 0.125 μg/g in feces and 0.100 μg/mL in plasma. All parameters fulfilled the validation criteria as described in the Supplementary Tables A,B and as given by the De Baere et al. (2012), the Veterinary International Conference of Harmonisation (VICH) guidelines (VICH, 2015) and the European Commission (EC) guidelines on the performance of analytical methods (Kehrenberg et al., 2004).

### Animal Experiment

During treatment with FF all animals presented a known treatment side-effect, namely diarrhea. For most animals this occurred during the second day of treatment and lasted for the entire treatment period. However, all animals were found to be clinically healthy during the entire treatment period. No control data is available to compare the extent of diarrhea with untreated animals.

#### Plasma FF Concentrations

**FIGURE 1:**
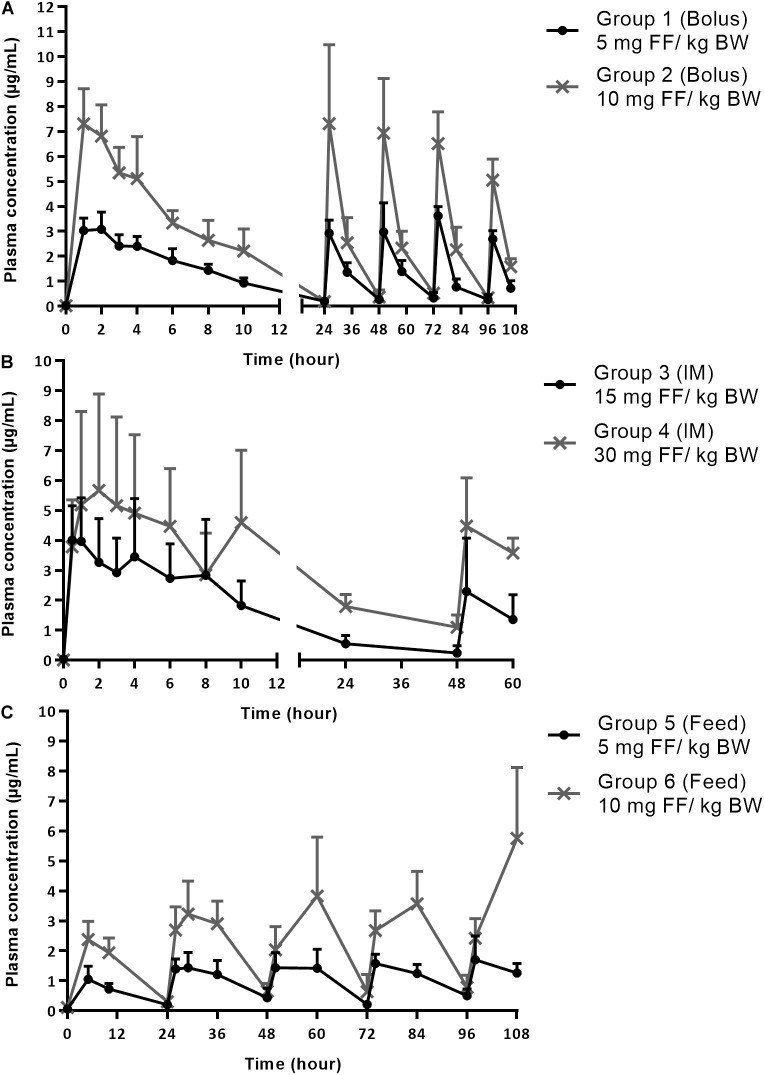
**(A)** Plasma concentrations of florfenicol (FF) after the first administration (0–24 h) and during entire treatment period (0–106 h) with bolus administration of 5 mg FF/kg BW in group 1 and 10 mg FF/kg BW in group 2. The bolus was administered once daily for 5 consecutive days. **(B)** Plasma concentrations of FF after the first administration (0–24 h) and during entire treatment period (0–58 h) with FF administered IM twice, with a 48 h-intervals in group 3 (15 mg FF/kg BW) and 4 (30 mg FF/kg BW). **(C)** Plasma concentrations of FF during entire treatment period (0–106 h) with medicated feed administered once daily at dosages of 5 mg FF/kg BW in group 5 and 10 mg FF/kg BW in group 6, for 5 consecutive days. All results are represented as mean + SD (*n* = 6).

The average plasma concentrations + standard deviation (SD) after FF administration are displayed in Figure 1A for treatment groups 1, 2 and Figure 1B for treatment groups 3, 4, up to 10 h after the first administration and during the full treatment period (106 h oral groups 1, 2, and 58 h for the IM groups 3, 4). Blood sampling is displayed in Figure 1C for the groups receiving medicated feed (5 and 6), during the full treatment period (106 h). The plasma PK parameters that were calculated for the different treatment groups are shown in Table 2. These parameters for FF in plasma are in agreement with other literature reports for IM administration (Dorey et al., 2017) and in-feed mixing of FF (Jiang et al., 2006).

**Table 2:** Overview of plasma pharmacokinetic (PK) parameters of florfenicol (FF): area under the curve from 0 to 24 h (AUC_0-24h_) and within this time frame calculated maximal plasma concentration (C_max_), and time of C_max_ (T_max_) for groups 1, 2, 3, and 4 after the first administration.

PK parameter	Group 1	Group 2	Group 3	Group 4	Group 5	Group 6
	PO bolus	PO bolus	IM	IM	PO feed	PO feed
	5 mg FF/kg BW	10 mg FF/kg BW	15 mg FF/kg BW	30 mg FF/kg BW	5 mg FF/kg BW	10 mg FF/kg BW
AUC_0-24h_ (h^∗^μg/mL)	25.2 ± 7.0^b^	53.9 ± 8.2^a^	44.7 ± 17.7^a^	86.3 ± 35.9^ab^	NA	NA
C_max_ (μg/mL)	3.4 ± 0.7^b^	7.4 ± 1.5^a^	4.8 ± 1.9^b^	6.0 ± 2.6^a^	NA	NA
T_max_ (h)	1.3 ± 0.5^a^	1.2 ± 0.4^a^	2.8 ± 2.9^a^	2.3 ± 1.2^a^	NA	NA
AUC_0-58h_ (h^∗^μg/mL)	NA	NA	75.1 ± 34.6	166.9 ± 35.4	NA	NA
AUC_0-106h_ (h^∗^μg/mL)	139.1 ± 30.3	296.4 ± 83.4	NA	NA	108.3 ± 23.7	231.5 ± 76.4
AUC_0-end_/D (h/mL)	1.0 × 10^-6^ ± 2.8 × 10^7a^	1.0 × 10^-6^ ± 3.4 × 10^7a^	1.4 × 10^-7^ ± 5.5 × 10^8a^	1.6 × 10^-7^ ± 2.7 × 10^8a^	7.2 × 10^-7^ ± 1.6 × 10^7a^	7.4 × 10^-7^ ± 2.4 × 10^7a^

*Also given are the AUC_*0*-*58 h*_ values for groups 3 and 4; equalling 2 injections with 48 h-interval for IM administration. And also the AUC_*0*-*106 h*_ values equalling 5 treatment days for oral (PO) administrations. The total values depicted as AUC_*0*-*end*_ (106 h oral groups, 58 h intramuscular (IM) were also normalized for dose (in μg) administered (AUC_*0*-*end*_/D). All data are represented as mean ± SD with *n* = 6 per group, for each calculated parameter. A difference in superscripts a, b denotes a statistical significant difference between these groups. Statistics were exerted using a single-factor ANOVA with a *post hoc* Tukey test (equal variances) or *post hoc* Games-Howell test (equal variances not assumed), significance level 0.05. NA, not applicable*.

Similar plasma concentrations were reached over a 24-h-timespan after the oral bolus administration of FF and the IM administration for groups 1 and 3, respectively in this study, despite the lower oral dosage. These findings are also in accordance with a study by Liu et al.(2003) indicating high oral bioavailability, in the range of 100%, of FF in pigs. Thus in spite of its low solubility in water, oral administration of FF can lead to high systemic availability. Moreover, lower C_max_ values were observed after IM administration in comparison with an oral bolus administration, which is likely due to a depot effect occurring in the muscle tissue and thus causing a prolonged slow release from the injection site, as also reported by Voorspoels et al. (1999) and Lobell et al (1994). The impact of in-feed mixing of FF on the oral bioavailability was also investigated by comparison of the AUC_0-106__h_ values between the groups receiving oral bolus (1,2) and medicated feed (5,6) with the same respective dosages. No statistical significant differences (*p* < 0.05) were detected between group 1 and 5 (*p* = 0.91) and group 2 and 6 (*p* = 0.30). Hence, the plasma concentrations of FF during the 5-day treatment period are not statistically different after oral bolus or medicated feed administration, for a given dose. Finally, the relation between dose and plasma concentration was linear. After normalizing the AUC_0-end_ values with the dose administered (in μg) for the sets of groups 1–2, 3–4, and 5–6, constant values between these sets of groups were achieved (Table 2). Hence, doubling or halving the dose of FF for the same administration route, will have a similar impact on systemic exposure to FF.

#### Intestinal and Fecal FF Concentrations

After lyophilisation of the intestinal and fecal samples, the loss of water content was calculated. Subsequently, the determined FF concentrations from the lyophilised material were compensated for based on the water loss from each individual sample. Hence, the FF concentrations as given throughout the manuscript are based on the weight of the actual samples, taking water content into account.

The intestinal concentrations in the different gastro-intestinal segments were compared over the different treatment groups (Table 3). Group 4, administered the highest dose of 30 mg FF/kg BW via IM injection, showed significantly higher (*p* < 0.05) FF concentrations in most of the gastro-intestinal segments (apart from ileum and colon) in comparison with the other groups. Group 6, administered medicated feed at 10 mg FF/kg BW, showed significantly higher jejunal concentrations in comparison with the other groups (apart from group 4). This is most probably related to the ongoing uptake of FF in the proximal segments, after medicated feed administration. The high FF concentrations established after IM administration are likely related to a mechanism of enterohepatic recirculation (Liu et al., 2003) and/ or a gastro-intestinal secretion from blood to gut lumen (De Smet et al., 2017), consequently these processes can also occur after oral administration of FF. However, neither of these mechanisms has been elucidated for FF in pigs. Furthermore, no significant differences were detected when comparing the intestinal concentrations between the other treatment groups over the different gastro-intestinal segments. These data suggest that the administration route has no effect on FF exposure in the gastro-intestinal tract. There is a dose-related influence, albeit not always statistically significant, with higher concentrations measured after administration of higher doses.

**Table 3:** Intestinal content concentrations of florfenicol (FF) in the different gastro-intestinal segments.

Mean	Group 1	Group 2	Group 3	Group 4	Group 5	Group 6
concentration	Bolus	Bolus	IM	IM	Feed	Feed
(μg/g)	5 mg FF/kg BW	10 mg FF/kg BW	15 mg FF/kg BW	30 mg FF/kg BW	5 mg FF/kg BW	10 mg FF/kg BW
^∗^Duodenum	22.9 ± 19.4^a^	39.5 ± 16.0^a^	52.2 ± 45.9^a^	285.8 ± 21.1^b^	60.8 ± 59.6^a^	255.3 ± 199.7^a^
	(*n* = 6)	(*n* = 4)	(*n* = 6)	(*n* = 4)	(*n* = 5)	(*n* = 4)
Jejunum	21.4 ± 6.2^a^	28.0 ± 22.8^a^	35.4 ± 14.8^a^	76.0 ± 24.6^b^	20.6 ± 20.4^a^	80.4 ± 33.0^b^
	(*n* = 6)	(*n* = 3)	(*n* = 6)	(*n* = 6)	(*n* = 6)	(*n* = 6)
Ileum	36.4 ± 19.8^a^	48.4 ± 31.3^a^	33.5 ± 39.6^a^	60.6 ± 43.1^a^	31.1 ± 20.8^a^	83.6 ± 51.8^a^
	(*n* = 45)	(*n* = 3)	(*n* = 5)	(*n* = 5)	(*n* = 4)	(*n* = 4)
Cecum	21.6 ± 13.5^a^	26.5 ± 12.5^a^	12.8 ± 6.3^a^	54.4 ± 12.9^b^	5.2 ± 4.0^a^	28.5 ± 23.0^a,b^
	(*n* = 6)	(*n* = 4)	(*n* = 5)	(*n* = 5)	(*n* = 3)	(*n* = 4)
Colon	19.5 ± 16.2^a^	20.1 ± 5.2^a^	16.1 ± 20.6^a^	31.5 ± 10.1^a^	13.3 ± 15.0^a^	11.8 ± 3.8^a^
	(*n* = 4)	(*n* = 4)	(*n* = 4)	(*n* = 3)	(*n* = 3)	(*n* = 5)

*With daily oral bolus administered in group 1 and 2 during 5 days. Groups 3 and 4 were administered two IM injections with 48 h-interval and groups 5 and 6 received medicated feed once daily during 5 days. Mean ± SD intestinal FF concentrations are displayed for the different segments. A difference in superscripts a, b denotes a statistical significant difference between these groups. Statistics were exerted using single-factor ANOVA with *post-hoc* testing (Tukey or Games-Howell^∗^ in case of unequal variances) and significance level *p* = 0.05*.

**FIGURE 2:**
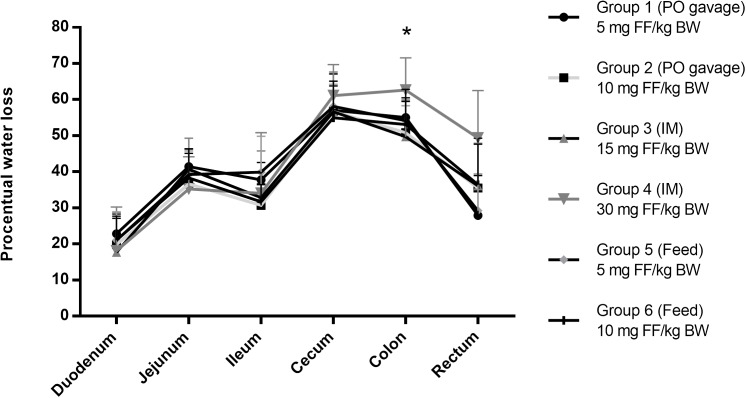
Water content calculated after a 48 h freeze-drying process of the intestinal content samples from all treatment groups. With the oral bolus groups 1 and 2 administered 5 and 10 mg FF/kg BW, respectively, groups 3 and 4 administered IM 15 and 30 mg FF/kg BW, respectively and groups 5 and 6 administered medicated feed at 5 and 10 mg FF/kg BW, respectively. Water content was calculated based on the sample weight pre- and post-freeze-drying. ^∗^Significant differences were detected between group 4 and group 1 (*p* = 0.030) after single-factor ANOVA with a *post-hoc* Tukey test (equal variances). No significant differences were detected for each of the other gastro-intestinal segments after a single-factor ANOVA with a *post-hoc* Tukey test (equal variances) or in case of the rectum segment a *post-hoc* Games-Howell test (equal variances not assumed), with significance level *p* = 0.05 for all tests.

FF concentrations in fecal samples were below the LOQ of 0.125 μg/g in all treatment groups. However, transitional diarrhea, a well-known side effect of FF in pigs, could potentially contribute to dilution of the samples yielding low fecal concentrations. The exact cause of this adverse effect is not known; although it is commonly hypothesized these effects are due to the irritable properties of the molecule, its bacterial killing properties causing the release of endotoxins or a shift in microbiota during therapy (i.e., microbial dysbiosis). For the latter it has been demonstrated in mice that an antimicrobial-induced dysbiosis is related to an impaired gastro-intestinal motility and an increase in fecal water content (Caputi et al., 2017). In the present study, pigs in all treatment groups developed diarrhea. The extent of diarrhea was estimated by comparing the weight loss from the intestinal and fecal samples pre- and post-freeze-drying (Figure 2). Normally, resorption of water is one of the main functions of the distal gastro-intestinal segments, which results in less water content. However, in this study the water content was highest in the cecum and colon segments in all treatment groups. After *post-hoc* comparison of the different groups, significant differences were detected (*p* > 0.05) in water content within the same intestinal segment; in group 4 (30 mg FF/kg BW) the colon segment showed significantly higher mean (*n* = 6) water content in comparison with group 1 (*p* = 0.030). In the other intestinal segments, significant differences were not detected.

In conclusion, this transient side-effect occurred during all treatment strategies applied in our experimental setup, meaning after 2 FF IM injections or after 5 days of one daily oral bolus or medicated feed administration, although a higher water content was observed for group 4 after IM administration of 30 mg FF/kg BW in cecum, colon and rectum.

### Microbiological Experiments and Intestinal Florfenicol Pharmacokinetics

The microbiological analysis of the fecal samples revealed a mean *E. coli* colony forming unit (CFU) count of 3.08 × 10^5^ ± 1.99 × 10^5^ per g faeces over the different treatment groups at time point 0 h (Supplementary Table C). At this time point, the vast majority of the isolates (99.5% up to 100%) were unable to grow on the FF supplemented MacConkey agar plates (16 μg FF/mL). Moreover, all identified isolates (3 colonies per plate) retained from the un-supplemented MacConkey agar had FF MIC values ranging between 2 and 16 μg/mL, while the isolates retrieved from MacConkey agar plates supplemented with FF (*n* = 3 colonies), all showed MIC values of ≥ 256 μg/mL (gradient test strip upper limit). However, at the end of the treatment period (58 h for IM groups, 106 h for oral groups), a clear shift was observed. While the total *E. coli* count on plain MacConkey hardly changed after treatment, with mean counts over the different treatment groups of 6.78 10^5^ ± 2.00 10^5^ CFU/g, these populations consisted mainly of isolates (from 86.8 up to 100.0%) able to grow on the FF supplemented MacConkey agar plates (Supplementary Table C). All isolates retrieved at time point 58 h or 106 h showed MIC values of ≥ 256 μg/mL. All randomly selected and purified colonies were confirmed as *E. coli* by MALDI-TOF MS analysis, with score values of ≥2.300.

**FIGURE 3:**
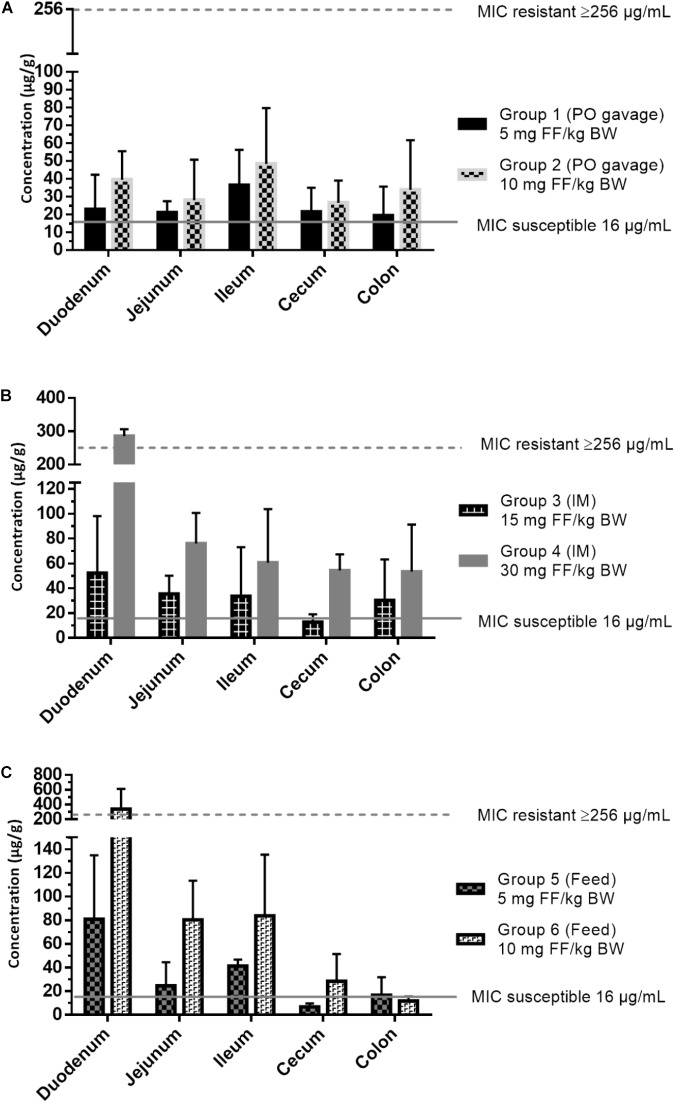
Intestinal content concentrations of florfenicol (FF) in different gastro-intestinal segments: duodenum, jejunum, ileum, cecum and colon at 10 h after the last administration, combined with minimal inhibitory concentration (MIC) values for susceptible and resistant *Escherichia coli* isolates. Results are presented as mean + SD (*n* = 6). **(A)** Group 1 (5 mg FF/kg BW) and 2 (10 mg FF/kg BW) administered an oral bolus of florfenicol (FF) during 5 days (once daily), **(B)** Group 3 (15 mg FF/kg BW) and group 4 (30 mg FF/kg BW) administered intramuscular FF injections twice (48 h interval), and **(C)** Group 5 (5 mg FF/kg BW) and 6 (10 mg FF/kg BW) administered medicated feed once daily during 5 days.

The bacteriological findings were subsequently combined with the PK data from the gastro-intestinal segments of the individual animals. Given the time-dependent (Watteyn et al., 2015) bacteriostatic effect of FF, the associated PK/PD index for efficacy is time above MIC in a 24 h dosing interval (T > MIC). However, because of the manner of intestinal sample collection, following euthanasia, a time-dependent gut concentration profile cannot be established over the whole course of therapy in this study. Hence, the intestinal concentrations measured in the different groups were matched with the wild-type MIC cut-off for susceptible isolates (16 μg/mL) and the gradient test strip upper limit for resistant isolates (256 μg/mL), as given in Figure 3. Data based on the PK/PD integration, complete eradication of susceptible *E. coli* strains in the different gastro-intestinal segments is predicted by the end of therapy, which was also reflected by the lack of susceptible isolates recovered from the fecal samples at the end of therapy. Furthermore, all resistant isolates retrieved at time 0 h and 58 h (end IM treatment) or 106 h (end oral treatment) displayed MIC values of at least 256 μg/mL, which is higher (apart from group 4) than the mean measured maximal FF concentrations ± SD (group 1: 36.4 ± 19.8 μg/g, group 2: 48.4 ± 31.3 μg/g, group 3: 52.2 ± 45.9 μg/g, group 4: 285.8 ± 21.1 μg/g, group 5: 60.8 ± 59.6 μg/g, group 6: 255.3 ± 199.7 μg/g), favoring selection of resistant isolates in all treatment regimens under FF pressure.

**FIGURE 4:**
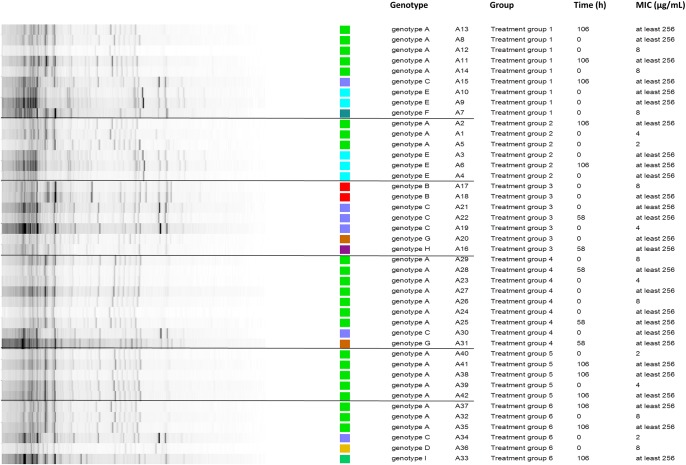
Repetitive element sequenced-based (rep)-PCR of 42 different retrieved Escherichia coli isolates (time point 0 h and also 58 h for intramuscular treated groups or 106 h for oral treated groups), from 6 different treatment groups and in total from 18 different pigs. The minimal inhibitory concentration (MIC in μg/mL) was determined for each isolate. With treatment group 1 and 2 administered an oral bolus at 5 and 10 mg florfenicol (FF)/kg BW, respectively, group 3 and 4 administered FF intramuscularly twice with 48-h interval at 15 and 30 mg FF/kg BW, respectively, and group 5 and 6 administered medicated feed at 5 and 10 mg FF/kg BW, respectively. Different genotypic groups or single isolates were found as indicated by the color scheme. The different groups are outlined between the black lines. Genotypic diversity occurred on group- and animal-level, hence excluding selection of one resistant mutant.

Based on earlier literature reports, FF resistance in *E. coli* is associated with values of 16 < MIC ≤ 512 μg/mL (White et al., 2000; Bischoff et al., 2002). Furthermore, the dissemination of FF resistance is mostly related to mobile genetic elements; with reports of different conjugative plasmids or transposons harboring FF resistance genes, which can be found in *E. coli* field strains (Doublet et al., 2005; Meunier et al., 2010). Hence, conjugative plasmids could contribute to the distribution of FF resistance genes in several bacterial species and in different animals (Cloeckaert et al., 2000; Kehrenberg et al., 2006). FF resistance can also be located on the chromosome or non-conjugative plasmids (Singer et al., 2004), which would still allow for selection of these resistant mutants under antimicrobial pressure. In our study, before and after treatment, multiple *E. coli* genotypes were detected in both the susceptible and resistant *E. coli* isolates (Figure 4). There was no obvious selection toward one or a limited number of genotypes after treatment, since the number of resistant genotypes seemed to increase after treatment. Even though the currently used methods cannot provide definite proof, the obtained results suggest that both selection of pre-existing resistant isolates and the horizontal transfer of resistance determinants toward originally susceptible strains may have contributed to the emergence of the resistant *E. coli* population, after treatment in this experimental setup. Finally, these findings were not related to an untreated control group. Hence, the spread of resistant genotypes from one treatment group to another cannot be excluded either.

Finally, in these experiments no effect of dose alteration or administration route on resistance selection in *E. coli*, as Gram-negative indicator bacterium, was observed. Even though the animals of group 4, administered the highest IM dose of 30 mg FF/kg BW, displayed elevated intestinal FF concentrations. In this sense, all microbiological experiments indicated a strong selection of resistant *E. coli* strains during treatment with FF, in all treatment groups. This may be related to the fact that all treatment strategies led to sufficiently high gastro-intestinal concentrations for eradication of susceptible strains and allowing for selective enrichment of resistant isolates.

## Conclusion

In general, a linear relationship between dose and FF plasma concentration was found for a given administration route. Following, no significant effects of the administration route on gastro-intestinal concentrations of FF were observed. However, in most gastro-intestinal segments, elevated FF concentrations were measured after IM administration of the highest dose, i.e., 30 mg FF/kg BW. No significant differences in FF intestinal concentrations were detected among the other treatment groups. Bacteriological analysis of the fecal samples demonstrated the presence of both FF susceptible and FF resistant *E. coli* isolates at the start of the treatment. The susceptible isolates displayed MIC values of 2–16 μg/mL. In relation to the high FF intestinal concentrations measured at 10 h after the last administration, eradication of susceptible *E. coli* strains is expected during treatment. All characterized resistant isolates displayed MIC values equal to or greater than 256 μg/mL. Hence, these high MIC values associated with FF resistance allowed for selection of resistance in the different gastro-intestinal segments, irrespective of treatment group.

These results are a cause for concern in terms for gastro-intestinal exposure of the microbiota to FF. Taking into account that the gut microbiota is a major resistance reservoir; exposure to such concentrations of FF could increase resistance selection and spread, as observed in the present study for *E. coli*. Furthermore, plasmid-mediated co-selection or cross resistance with FF also has to be contemplated. FF is used for respiratory tract infections, hence the effects of the molecule on the microbiota could be considered as an adverse event in terms of AMR. However, awareness of this adverse event needs to be implemented, in order to warrant a prudent use of FF in respiratory tract infections and minimize the risk of AMR selection and spread from the microbiota.

## Author Contributions

JDS, SC, FB, FH, PDB, and MD prepared the experimental design. JDS conducted the animal experiments, performed the analytical method development and sample analysis. JDS, FB, and GR performed the bacteriological analysis. JDS and MD performed the pharmacokinetic analysis. JDS did the statistical analysis. JDS, SC, FB, GR, FH, PDB, and MD prepared the manuscript. All authors read and approved the final manuscript.

## Conflict of Interest Statement

The authors declare that the research was conducted in the absence of any commercial or financial relationships that could be construed as a potential conflict of interest.
