# Supplementary material for: Similar Gastro-Intestinal Exposure to Florfenicol After Oral or Intramuscular Administration in Pigs, Leading to Resistance Selection in Commensal Escherichia coli
Source: Front Pharmacol. 2018 Nov 6;9:1265. doi: 10.3389/fphar.2018.01265 (PMC6232271; doi:10.3389/fphar.2018.01265)
Supplement: Supplementary file 1 [file Table_1.DOCX]

1. **Supplementary files**

Validation parameters for the analysis of florfenicol using an UPLC-PDA method

| Table A. Main validation parameters: linearity (with correlation coefficient r and goodness-of-fit coefficient g), limit of quantification (LOQ) and limit of detection (LOD) for florfenicol (FF) in manure (µg/g) and plasma (µg/mL). | | | | |
| --- | --- | --- | --- | --- |
| **Matrix** | r | g | LOQ (µg/g or µg/mL) | LOD (µg/g or µg/mL) |
| Manure | 0.9986 | 5.10 | 0.126 ± 0.0062 | 0.061 |
| Plasma | 0.9993 | 5.29 | 0.088 ± 0.0072 | 0.0013 |
|  | | | | |

| Table B. Validation results for the parameters accuracy and precision based on different theoretical concentration levels for florfenicol (FF) in manure (µg/g) and plasma (µg/mL). | | | | |
| --- | --- | --- | --- | --- |
| **Matrix** | Theoretical concentration  (µg/mL or µg/g) | Mean concentration ± SD  (µg/mL of µg/g) | Precision  RSD (%) | Accuracy  (%) |
| **Manure** | 0.125^a^ | 0.126 ± 0.00621 | 4.9 | 0.9 |
|  | 2.50^a^ | 2.532 ± 0.0332 | 1.3 | 1.3 |
|  | 25.00^a^ | 25.013 ± 1.370 | 6.8 | -1.0 |
|  | 2.50^b^ | 2.584 ± 0.230 | 8.9 | 3.4 |
|  | 25.00^b^ | 24.744 ± 0746 | 3.0 | -1.0 |
| **Plasma** | 0.10^a^ | 0.0857± 0.00659 | 7.7 | -14.3 |
|  | 2.50^a^ | 2.628 ± 0.149 | 5.7 | 5.1 |
|  | 25.00^a^ | 27.004 ± 2.126 | 7.9 | 8.0 |
|  | 2.50^b^ | 2.544 ± 0.0943 | 3.7 | 1.8 |
|  | 25.00^b^ | 25.241 ± 2.326 | 9.2 | 1.0 |
| ^a^ Within-run accuracy and precision (n=6)  ^b^ Between-run accuracy and precision (n=6)  SD: standard deviation; RSD: relative standard deviation.  Acceptance criteria: *accuracy*: > 0.01 µg/mL or µg/g: −20% to +10%; *within-run precision*: *RSD_max_*: ≥ 0.01 µg/mL or µg/g and < 0.1 µg/g or µg/mL: 15%, ≥ 0.1 µg/mL or µg/g: 10%; *between-run precision*: RSD_max_ = 2^(1-0.5logC)^ (with C the concentration (10^-9^) at which the samples were spiked). | | | | |

| Table C: Bacteriological analysis of faecal samples at time point 0 h before florfenicol (FF) treatment and at the end of treatment (58 h IM groups, 106 h oral groups) from three random selected pigs from each treatment group with colony counts (CFU/g) ± standard deviation (SD) performed on MacConkey (MC) agar and MC supplemented with FF at 64 µg/mL (EUCAST ECOFF). Following resistance percentages were also calculated by the formula: (CFU/g MC + FF)/ (CFU/g MC) x 100. | | | | | | |
| --- | --- | --- | --- | --- | --- | --- |
| **Colony count**  **CFU/g (n=3)** | **Group 1**  PO  10 mg FF/kg BW | **Group 2**  PO  5 mg FF/kg BW | **Group 3**  IM  15 mg FF/kg BW | **Group 4**  IM  30 mg FF/kg BW | **Group 5**  Feed  10 mg FF/kg BW | **Group 6**  Feed  5 mg FF/kg BW |
| Total count  0 h | 3.9 10^5^ ± 3.5 10^5^ | 5.9 10^5^ ± 1.1 10^5^ | 5.4 10^5^ ± 5.5 10^4^ | 5.5 10^5^ ± 2.2 10^5^ | 5.0 10^5^ ± 5.3 10^3^ | 4.5 10^5^ ± 2.8 10^5^ |
| MacConkey + FF 0 h | 1.7 10^2^ ± 2.9 10^2^ | 2.5 10^2^ ± 3.5 10^2^ | 2.5 10^3^ ± 1.6 10^3^ | 2.5 10^2^ ± 2.9 10^2^ | 0.0 | 0.0 |
| Resistance % | 0.04 | 0.04 | 0.5 | 0.05 | 0.0 | 0.0 |
| Total count  58 h or 106 h | 6.8 10^5^ ± 1.4 10^5^ | 6.4 10^5^ ± 7.4 10^4^ | 9.0 10^5^ ± 6.5 10^4^ | 6.5 10^5^ ± 9.6 10^4^ | 8.4 10^5^ ± 1.4 10^5^ | 7.2 10^5^ ± 1.9 10^5^ |
| MacConkey + FF  58 h or 106 h | 6.8 10^5^ ± 1.0 10^5^ | 5.8 10^5^ ± 3.2 10^4^ | 8.1 10^5^ ± 8.1 10^4^ | 5.6 10^5^ ± 2.3 10^4^ | 8.0 10^5^ ± 7.6 10^4^ | 7.6 10^5^  ± 1.2 10^5^ |
| Resistance % | 100.0 | 91.3 | 90.0 | 86.8 | 95.6 | 100.0 |
